# Effect of Seawater with Average Salinity on the Moisture Content, Ash Content and Tensile Strength of Some Coniferous Wood

**DOI:** 10.3390/ma16082984

**Published:** 2023-04-09

**Authors:** Kamil Roman, Emilia Grzegorzewska, Mateusz Leszczyński, Seweryn Pycka, Jan Barwicki, Ewa Golisz, Patrycja Zatoń

**Affiliations:** 1 Institute of Wood Sciences and Furniture, Warsaw University of Life Sciences, 166 Nowoursynowska St., 02-787 Warsaw, Poland; kamil_roman@sggw.edu.pl (K.R.); s196970@sggw.edu.pl (M.L.); s190114@sggw.edu.pl (S.P.);; 2 Department of Rural Technical Infrastructure Systems, Institute of Technology and Life Sciences, National Research Institute, 05-090 Raszyn, Poland; jbarwicki@gmail.com; 3 Institute of Mechanical Engineering, Warsaw University of Life Sciences, 164 Nowoursynowska St., 02-787 Warsaw, Poland; ewa_golisz@sggw.edu.pl

**Keywords:** mechanics, the tension of wood, stresses, soaking in salted water, tensile strength along the fibres, ash content measurement, coniferous wood

## Abstract

In this paper, the differences in mechanical strength tested during the static tensile and compression test of Scots pine (*Pinus sylvestris* L.), European larch (*Larix decidua*) and Norway spruce (*Picea abies*) wood exposed to continuous soaking in water with a salinity of 7‰ were determined. The value of salinity corresponded to the average salinity on the Polish Baltic coast. This paper also aimed to examine the content of mineral compounds absorbed during four cycles of two weeks each. The essence of the statistical research was to identify the effect of the mineral range compounds and salts depending on the mechanical strength of the wood. Based on the results of the experiments, it can be concluded that the medium used has a specific effect on the wood species’ structure. The effects of soaking on the wood parameters depend obviously on the type of wood. A tensile strength test of pine, as well as the tensile strength other species, was enhanced by incubating it in seawater. A native sample’s initial mean tensile strength was 82.5 MPa, which increased to 94.8 MPa in the last cycle. It was found that the larch wood had the lowest tensile strength difference (9 MPa) of the woods studied in the current study. Four to six weeks of soaking was necessary to notice an increase in tensile strength.

## 1. Introduction

Wood can absorb water in the form of liquid while in contact with it or as vapor from the atmosphere [1]. Due to its hygroscopicity, wood, as part of a living tree as a material, always shows some moisture content [2,3]. Moisture affects all the wood properties [4]. Moisture in cell cavities only increases the weight. The moisture content retained in cell walls ranges from about 20 to 40 percent [5]. The theoretical point where the cell walls are completely saturated and the cell lumen is empty is known as the fibre saturation point [1]. Above this point the moisture penetrates cavities, and as soon as they are filled up, the maximum moisture content the wood can retain is reached. The maximum, depending on density, can be very high or low. For example, balsa can retain even 800 percent moisture, pine 250 percent and beech 120 percent [6]. Moisture results in wood shrinkage or swelling. We can differentiate between two states of wood protection: a dry protective state, where the wood moisture is below 20% [7], and a wet protective state with a wood moisture above 80% [8].

Moisture loss results in shrinkage and swelling. Typically, the measurement changes are anisotropic. They differ in the axial, radial and tangential directions. A varied shrinkage and swelling in different growth directions is mostly attributed to the cell wall anatomy. The general factors responsible for shrinkage and swelling are the content of extracts, mechanical stresses and wood structure irregularities. The higher the wood density, the higher the wood shrinkage and swelling as denser (heavier) wood shows a higher cell wall moisture. Natural extracts decrease shrinkage and swelling as they occupy spaces in cell walls that otherwise could be taken by water. Mechanical stresses (compression or tension) can result in permanent wood cell deformations that, in turn, affect shrinkage and swelling [9,10].

Saltwater can cause many damages to softwood, including affecting its dimensional stability and initiating osmosis process. Meanwhile, the salt content in seawater can influence the corrosion of metal components connecting the wood [11,12]. Studies conducted on pine wood have shown that prolonged exposure of wood to saltwater can increase the acid content in the wood and affect its strength [11]. It has also been indicated that the salt content level in saltwater influences the osmotic effect and induces different reactions in various wood species [13].

This study aimed to analyze the effect of salt water on the mechanical parameters of wood species used in industries in contact with seawater. It was determined whether coniferous wood species, such as pine *(Pinus sylvestris* L.), larch (*Larix decidua*) and spruce (*Picea abies*), show the specific parameters of wood with a simultaneous effect of seawater and time based on the tensile strength of the material. Measuring the parameters will define the material analysed in terms of its use. Further, in this study the samples were incinerated to identify a change in the content of salt and other minerals. Scots pine wood is used in various industries such as construction, furniture and paper production. It is widely used as a species known for its lightweight softwood, which is easy to work with and has a variety of applications.

## 2. Methodology

### 2.1. Material

**Figure 1:**
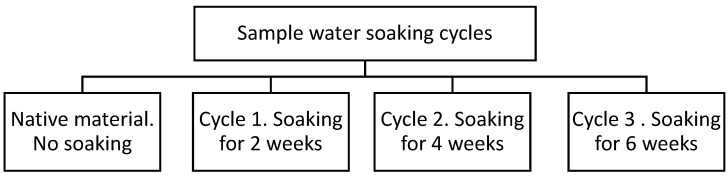
Number and duration of soaking cycles of wood samples in water.

The material was acquired from the coastal strip, which was up to 50 km away from the coastline; the Gościno forest inspectorate, Dębica forest district. An extremely important value of wood in the sectors using wood in contact with the sea is susceptibility to processing [14]. Twelve tensile samples were formed from the material for each type of wood, and 3 types of wood were used for testing, which gives a total of 36 tensile samples. The samples were placed in a 60 L aquarium filled with distilled water and sea salt. For the container, at an adequate ratio, salt was mixed with water with the share of 7‰ (7 g of salt per 1000 g of water) [15]. The raw material was purchased in a specialized chemical store, and the salt consisted entirely of sodium chloride. The samples were divided depending on the material type. The samples were subjected to 4 soaking periods; in each cycle 9 samples were used—3 samples for pine, 3 for spruce and 3 for larch. The number and length of particular periods of sample soaking in water are presented in Figure 1.

### 2.2. Material Moisture Measurement

In selected periods, compliant with the research methodology, the samples were removed from the container in cycles. Once taken out from the container, the samples were dried in a lab chamber at a temperature of 105 °C [9]. The samples were further placed in a lab cuvette to standardize the moisture for all the samples measured. A moisture of 12% was recorded after two weeks [5,16,17]. The calculation methodology is explained in Equation (1). After that time, their weight was measured to verify the moisture, applying the absolute moisture formula. The moisture (*M*) of the wood samples was investigated for the original material distribution. The value was determined according to Formula (1).

(1) $$M=\frac{m_{w}-m_{s}}{m_{w}}\cdot 100\%$$

*m_w_*—weight of wet wood samples.

*m_s_*—weight of wood samples dried to 0% moisture.

The use of a lab drier facilitated plotting compliant with the parameter guidelines in the applicable literature [18,19]. Reaching the moisture required sample drying in the drier chamber. As for the present studies, the moisture of the samples of crushed wood material was assayed compliant with the PN-ISO 589:2006 [18] guidelines. Prior to entering the condensing chamber, the material’s moisture was checked again. Randomly selected 5 g samples were tested. The studies were performed with the Radwag moisture analyser MAC 50 type (Radom, Poland).

### 2.3. Tensile Testing

**Figure 2:**
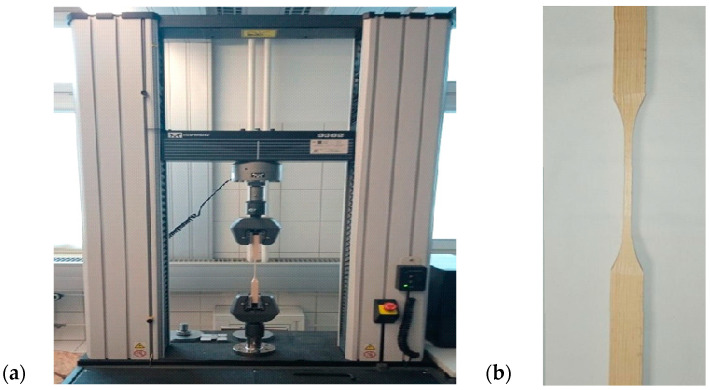
(**a**) Test stand with suction tensile holders for tensile testing; (**b**) samples used for tensile testing.

Lab tensile testing along the fibres was conducted with the testing machine Instron 3382 (Norwood, MA, USA). The suction jaw holder was test-adjusted. The stand included a testing machine, a set of tensile testing clamps, an auxiliary device and a measurement PC with Instrom IX software. For the test stand with suction tensile holders, see Figure 2.

Tensile testing is a basic test to determine the mechanical properties of materials [20,21]. The test measures the characteristics to be applied to identify the tensile strength, flow stress and strain energy of the sample. Tensile testing was performed in compliance with ISO 527-1 [22]. The statistical analysis of the results was carried out with the analysis of variance (ANOVA). Following the methodology, to identify whether the samples belonged to homogenous groups, a post-hoc Duncan’s test was applied.

### 2.4. Ash Content Measurement

The material sample was incinerated to assay the ash content. The ash content is related to the mineral saturation, as iron, silicon, aluminum, magnesium, calcium, sodium and potassium can be assayed [23]. The ash content was identified with the method of slow incineration in a muffle furnace. According to the literature, the preparation of the sample required drying it. The weighed sample was placed in the crucible. The weighed amounts prepared in three cycles (the weight measurement accuracy was 0.0001 g) were about 2 g per sample. The samples were incinerated in the muffle furnace, earlier heated up to 805 °C [24,25]. It took about two hours to assay the ash content. The crucible-incinerated sample was placed in the desiccator to cool down. Then the crucible weight together with ash was determined. The statistical analysis of the results was performed with the analysis of variance (ANOVA). The method facilitated determining the effect of the parameters measured in the successive soaking cycles.

## 3. Results

### 3.1. Moisture

**Figure 3:**
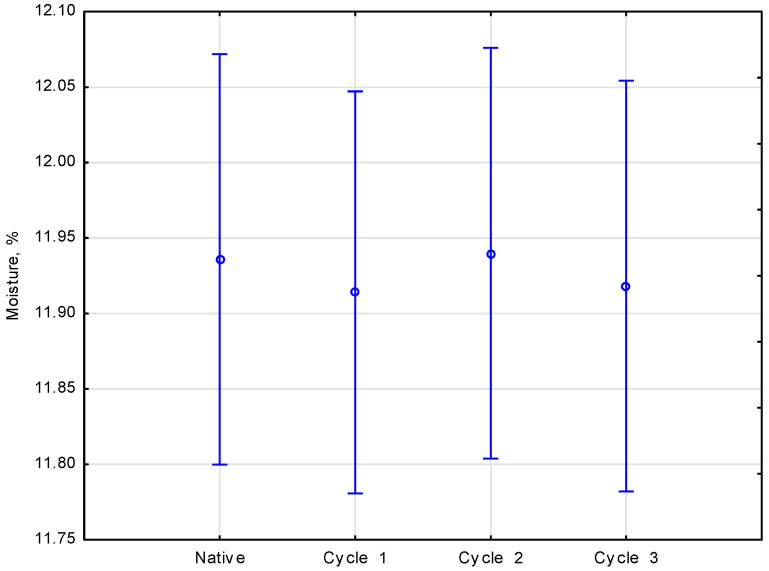
Effect of the number of salinated water soaking cycles applied on wood moisture.

Moisture is one of the primary parameters considered when investigating wood [17]. The moisture content of pine, spruce and larch samples was referred to as the sample soaking cycle. A breakdown of the means of the analysis of the effect of the number of salinated water soaking cycles applied on wood moisture is presented in Figure 3.

The statistical analysis [26] of the research results was conducted with the use of the one-way analysis of variance. The method facilitated determining the effect of the selected parameters on soaking cycles. The number of experiments used to calculate the mean value and the error bar of the moistures was three repetitions, which resulted in 36 analysis values. The statistical analysis demonstrated no significant differences between the parameters measured, which was confirmed with the value of significance *p* = 0.99097, for the value of empirical statistics *F*(3.89) = 0.03552. It was noted that the moisture did not exceed 12%.

### 3.2. Strength Testing

After a static tensile test was performed compliant with the applicable norms, the results were provided in tables. The lowest tensile strength for pine was 62.8 MPa and the highest was 89.7 MPa. The mean strength for all the samples measured was 82.5 MPa. The lowest strength for spruce was 78 MPa and the highest was 136.1 MPa. The mean strength for all the spruce samples measured was 98.8 MPa. The lowest strength of larch was 65.3 MPa, while the highest was 88.9 MPa and the mean strength for all three samples was 86.3 MPa. The results of tensile testing along the fibres in pine, spruce and larch samples for native material (non-soaked samples) are provided in Table 1.

Tensile testing along the fibres was performed for cycle 1 pine samples, soaked in the prepared conditions for two weeks. The lowest strength for pine samples was 80 MPa, while the highest was 85.6 MPa, and the mean strength was 82.8 MPa. The lowest spruce strength was 80 MPa and the highest was 132.4 MPa, while the mean strength of all the three samples was 99.1 MPa. The lowest strength recorded for larch was 80.9 MPa, while the highest was 100.6 MPa, and the mean strength of all the three samples was 88.5 MPa. The results of tensile testing along the fibres for the samples of the respective first-cycle materials are presented in Table 2.

The tensile testing along the fibres involved cycle two samples prepared by soaking the material for 4 weeks in a specially prepared solution. It was determined that the lowest value for pine samples was 77.9 MPa, while the highest was 95.9 MPa, and the mean strength of all three samples was 87.6 MPa. The lowest strength of the spruce samples was 72.4 MPa, while the highest was 135.4 MPa, and the mean strength of all three samples was 100.6 MPa. The lowest larch strength was 58.7 MPa, whereas the highest was 104.1 MPa, and the mean strength of all three samples was 93.9 MPa. The results of the tensile testing along the fibres of all the samples soaked for four weeks are presented in Table 3.

The successive testing stage identified the tensile strength of the cycle 3 samples along the fibres, soaked for six weeks. The lowest strength of pine samples was 78.3 MPa and the highest was 114.5 MPa. The mean strength of pine samples was 94.8 MPa. The lowest strength of cycle three spruce samples was 100.5 MPa and the highest was 120.6 MPa. The mean strength of all the spruce samples measured was 113.8 MPa. The lowest strength of larch was 87.1 MPa and the highest was 105.3 MPa. The mean strength of the larch samples was 95.3 MPa. The results of tensile testing along the fibres of the respective third cycle materials are given in Table 4.

**Figure 4:**
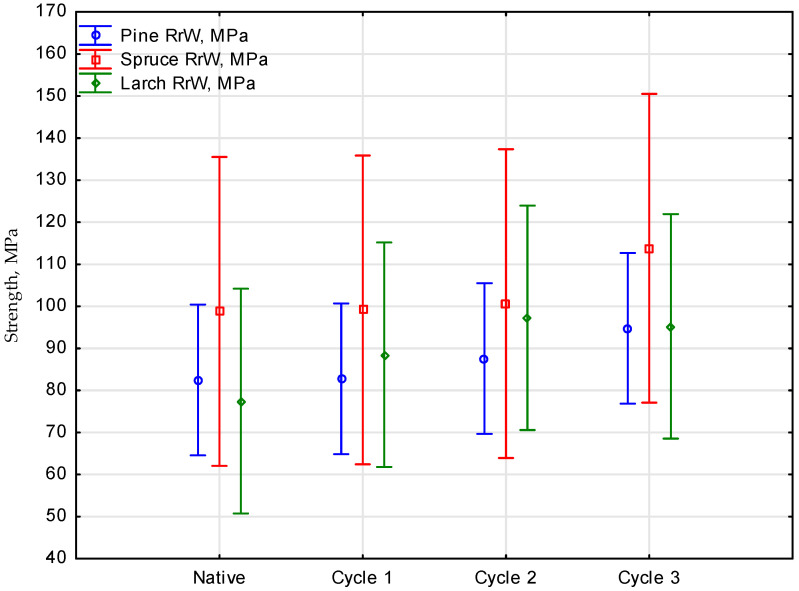
Effect of the number of salinated water soaking cycles on the tensile strength values of a given wood species.

The method facilitated determining the effect of tensile strength parameters of the samples measured in successive cycles. The statistical analysis has demonstrated no significant differences among the parameters, which was confirmed with the value of significance *p* = 0.91931, for the values of empirical statistics *F*(9, 14.753) = 0.39370. It was observed that the sample strength, although slightly, was increasing with the number of cycles for all the materials. The number of experiments used to calculate the mean value was three repetitions, which resulted in 36 analysis values. A breakdown of the means of the analysis of the effect of the number of soaking cycles applied in salinated water on the tensile strength values for a given wood species is provided in Figure 4.

As for the statistical analysis of strength parameters for pine tested in a few cycles, the level of significance *p* was higher than the assumed significance level alpha equal to 0.05. This means that the mean effects of the analysis of the number of soaking cycles on the tensile strength values for pine did not show any considerable differences. In that case, only one homogenous group was created with all the values for pine soaking cycles.

As for the statistical analysis of strength parameters of the spruce samples prepared and tested in a few cycles, the level of significance *p* was higher than the assumed significance level alpha equal to 0.05. The effect of the number of spruce soaking cycles on tensile strength values did not show any significant differences. Similarly, as in the earlier one, the post-hoc test demonstrated only one homogenous group with all the least squares means for the soaking cycles measured.

In the last case of the statistical analysis of tensile strength parameters of the larch samples tested in a few cycles, the level of significance *p* was higher than the assumed level of significance alpha equal 0.05.

### 3.3. Ash Content

The last stage of the study was assaying the ash content. The studies analysed the percentage of ash content in the pine, spruce and larch samples in the cycles prepared. The samples were placed in the crucibles ranging from 1.75 to 2.36 g (the mean sample weight was 1.8 g) and incinerated in the muffle furnace compliant with the methodology in a sufficient number of replications. A table breakdown of the percentage share of ash in native material samples is given in Table 5.

An interesting aspect was determining the effect of the medium in the form of salt solution on the ash content in the wood sample. The results of the study have been broken down into tables with the percentage content of ash in specific cycles for a given tree species. For native material, no significantly high changes were noted where the ash content was, on average, about 0.5%. Native material larch, similar to pine, showed an ash content below 0.5%. In cycle 1 the highest ash share was recorded for pine, while the extreme result for one of the samples was 0.9%. The other samples also slightly increased the mineral content. In cycle 2, the ash content in pine increased and it was almost 1%. The other samples did not change, maintaining their mineral content compared to cycle 1. In cycle 3, the mineral content for all the samples was similar, accounting for about 0.7%. Probably pine decreased its mineral content as mineral supersaturation was recorded in cycle 2. As for the other species, the supersaturation effect could be noted already at the beginning of the cycle 1 treatment.

**Figure 5:**
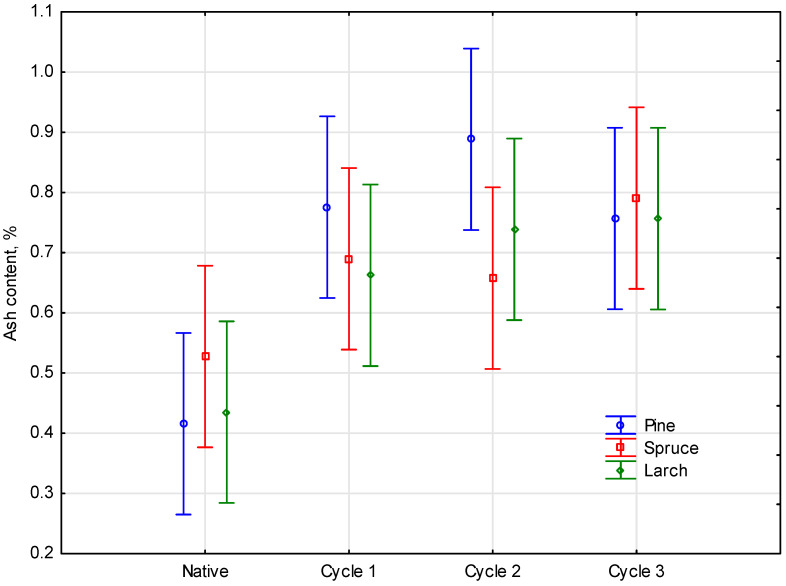
Effect of the number of salinated water soaking cycles on the ash content in respective wood species.

The statistical analysis identified a slight occurrence of significant differences between the parameters, which was confirmed with the level of significance *p* = 0.40470, for empirical statistics F(6, 24) = 1.0750. It was noted that the ash content, although inconsiderably (a difference of about 0.5%), was increasing together with the number of cycles for all the materials measured. A breakdown of the means of the analysis of the effect of the number of soaking cycles in salinated water on the ash content of a given tree species is provided in Figure 5.

Compliant with the methodology, to make a breakdown into respective homogenous groups, a post-hoc Duncan’s test was performed. As for the statistical analysis of the wood parameters assayed in a few cycles, the level of significance *p* was lower than the mean significance level alpha equal to 0.05. This means that the mean effects of the number of soaking cycles on the absorption of mineral values of the respective wood species differed slightly. In that case, a few homogenous groups were identified and attributed to overlapping squares means of the parameter assayed.

## 4. Discussion

In light of the experimental studies, it can be stated that the medium affected the structure of the wood species in a specific way. The wood treatment method influences not only the strength but also the absorbance of mineral materials. The effect of soaking on the physical parameters depended on the wood species.

Analysing the results of tensile testing along the wood fibres, it was identified that the highest strength was that of spruce. The mean strength demonstrates that the strength of that species increased. With every cycle, the strength was increasing continuously until the last cycle, exceeding 110 MPa. The material showed the highest difference in strength between the last and the first batch (15 MPa). A considerable increase in strength can be observed only after four and six weeks.

Pine, similarly to the other species, was also susceptible to soaking in seawater, which enhanced the result of tensile testing along the fibres. The initial mean value of native samples was 82.5 MPa and it was similar to the value reported in the literature [27,28]. In the last cycle, the mean strength increased to 94.8 MPa. The difference between the strength values was 12.3 MPa. In that case, similarly to in spruce, an increase in strength could be observed in the samples soaked from four to six weeks.

According to the results of the present study, larch was the wood with the lowest difference in strength (9 MPa). The initial mean value determined in the static tensile testing was 86.3 MPa, increasing the value together with the number of cycles up to 95.3 MPa. Thus, for larch wood, similarly to the other species, an increase in strength could be noted only in the samples soaked for four to six weeks.

The ash content, despite inconsiderable overlapping homogenous groups, showed slight differences in the ash content across the wood species [29]. The highest share occurred across the species in various cycles and, for pine, it was the second cycle; for spruce, the first cycle; and for larch, the third cycle. The content in each species and for each cycle exceeded one percent in one case only: for the second replication of the spruce sample for cycle 3. For the rest of the samples, the percentage content of ash ranged from 0.34% in native material to around 0.9% reported in further cycles, when soaked in a specially prepared saline solution.

When analyzing the strength results, a slight improvement in the tensile strength results for each wood species can be seen with subsequent soaking cycles, so it can also be assumed that the strength parameters will improve. This can be used as another method of chemically modifying wood. According to research conducted at the University of Eastern Finland, soaking pine wood in low-salt water for two weeks can reduce the content of lignin and hemicellulose in the wood, increasing its susceptibility to impregnation and thereby improving its resistance to external conditions. In addition, short-term soaking of pine wood in water can reduce its hardness, making it easier to work with and potentially increasing its attractiveness for some artistic applications [30].

## 5. Conclusions

The study shows that the mechanical properties of wood soaked in seawater are enhanced; however, they do not go exceptionally beyond the Polish norm. An interesting aspect is spruce wood strength, which should be the highest of all the species tested, including the native sample. In this research, samples of pine, spruce and larch were tested for tensile properties. The results showed that for all the materials, the strength increased slightly with the number of cycles. According to the statistical analysis, the parameters measured in successive cycles did not show any significant differences, in terms of statistical significance, as confirmed by the data. All the samples had mean strengths between 58.7 MPa and 135.4 MPa. The strongest mean strength values were found in the spruce sample, which was the most diverse. In summary, the results of the experimental studies show that the type of medium used as well as the method of wood treatment had a significant impact on the strength and structural characteristics of the wood species. It was found that spruce showed the highest strength with an ongoing increase in strength with each cycle. It was found that pine and larch, as well, showed strength increases between four and six weeks after soaking them. The strength results for each wood species improved slightly with subsequent treatment cycles; however, this did not occur for all wood species.

As part of the study, the effects of the salt solution medium on the wood samples’ ash content in different drying cycles were also examined. It has been determined that there has been a slight increase in the ash content, around 0.5%, combined with an increase in the number of cycles, which has been seen for all the materials measured in the experiment. The highest ash content was found in pine in cycle 2, nearly 1%, but it decreased in cycle 3, possibly due to mineral saturation. The ash content of larch and pine trees was the lowest in native material and was below 0.5%. According to the statistical analysis, there were slight but significant differences between the parameters of these trees. Depending on which type of wood species was used, there was a slight difference in the ash content. The process of soaking wood in low-salt water for two weeks can also reduce the amount of lignin and hemicellulose in the wood, making it easier to work with and improving its resistance to external forces. The development of the test stand required accuracy and diligence to facilitate reproducing the conditions wood is exposed to. No visible structural defects were found. However, the parameter that could disturb the simulation of the test stand was the fact of no water change or no water disturbance. Those two actions to some extent limited the absorption of mineral values by the wood material tested as soil crystals could have settled on the surface and not on the wood structure. The study results were verified with statistical analyses, which identified a correlation between the wood strength and the effect of mineral values. This means that wood absorbed a considerable amount of mineral salts which, at the final stage, enhanced the wood strength.

## Figures and Tables

**Table 1:** Tensile testing along the fibres of respective materials for native material.

Material	Cross-Sectional Dimensions, mm		Sample Destructive Force, kN	Tensile Strength along the Fibres, MPa
	A	B	F	RrW
Pine	18	4.42	7.56	95.0
	17.95	3.95	6.36	89.7
	17.62	4.22	4.67	62.8
Spruce	17.34	4.32	5.84	78.0
	17.7	4.6	6.7	82.3
	18.1	4.1	10.1	136.1
Larch	18.5	4.24	5.12	65.3
	18.8	3.9	5.74	78.3
	20.04	4.2	7.48	88.9

**Table 2:** Tensile testing along the fibres of respective cycle 1 materials.

Material	Cross-Sectional Dimensions, mm		Sample Destructive Force, kN	Tensile Strength along the Fibres, MPa	Sample No.
	A	B	F	RrW	
Pine	18.5	4.24	6.49	82.7	58
	19.27	4.22	6.96	85.6	59
	18.9	4.5	6.80	80.0	60
Spruce	19.01	4.9	7.92	85.0	70
	19.5	5.01	7.82	80.0	71
	19.89	3.95	10.4	132.4	72
Larch	19.78	4.6	9.15	100.6	82
	19.45	4.87	7.95	83.9	83
	18.09	4.91	7.19	80.9	84

**Table 3:** Tensile testing along the fibres of respective cycle 2 materials.

Material	Cross-Sectional Dimensions, mm		Sample Destructive Force, kN	Tensile Strength along the Fibres, MPa
	A	B	F	RrW
Pine	19.5	4.8	7.287	77.9
	18.06	4.65	7.48	89.1
	18.27	3.98	6.97	95.9
Spruce	18.09	4.67	6.12	72.4
	18.5	4.87	12.2	135.4
	18.4	4.9	8.48	94.1
Larch	18.2	4.86	5.19	58.7
	19.05	3.1	7.62	129.0
	18.9	4.56	8.97	104.1

**Table 4:** Tensile testing along the fibres of respective cycle 3 materials.

Material	Cross-Sectional Dimensions, mm		Sample Destructive Force, kN	Tensile Strength along the Fibres, MPa
	A	B	F	RrW
Pine	17.98	4.14	8.52	114.5
	18.9	4.8	7.1	78.3
	18.56	4.2	7.14	91.6
Spruce	17.6	4.6	9.74	120.3
	19.45	3.81	8.94	120.6
	19.3	4.59	8.9	100.5
Larch	20.3	4.69	8.29	87.1
	20.45	4.82	9.21	93.4
	17.8	4.9	9.18	105.3

**Table 5:** Breakdown of the percentage share of ash.

Material	Cycle	Sample Weight, g	Ash Weight, g	Ash Content, %	Average Ash Content, %
Pine	0	1.763	0.009	0.51	0.416 ^a^
		2.358	0.008	0.34	
		1.764	0.007	0.40	
Spruce		1.764	0.011	0.62	0.527 ^a,b,c^
		1.764	0.009	0.51	
		2.003	0.009	0.45	
Larch		1.762	0.009	0.51	0.435 ^a,b^
		1.762	0.007	0.40	
		1.762	0.007	0.40	
Pine	1	1.763	0.013	0.74	0.776 ^d^
		1.761	0.012	0.68	
		1.763	0.016	0.91	
Spruce		1.763	0.012	0.68	0.690 ^c,d^
		1.761	0.013	0.74	
		1.999	0.013	0.65	
Larch		1.76	0.008	0.46	0.662 ^b,c,d^
		1.762	0.012	0.68	
		1.762	0.015	0.85	
Pine	2	1.761	0.017	0.97	0.888 ^d^
		1.764	0.014	0.79	
		1.766	0.016	0.91	
Spruce		1.762	0.012	0.68	0.658 ^b,c,d^
		1.748	0.012	0.69	
		1.985	0.012	0.61	
Larch		1.758	0.012	0.68	0.739 ^c,d^
		1.76	0.015	0.85	
		1.763	0.012	0.68	
Pine	3	1.763	0.012	0.68	0.757 ^c,d^
		1.761	0.013	0.74	
		1.764	0.015	0.85	
Spruce		1.765	0.008	0.45	0.791 ^d^
		1.764	0.018	1.02	
		2.003	0.018	0.90	
Larch		1.762	0.013	0.74	0.756 ^c,d^
		1.761	0.014	0.80	
		1.765	0.013	0.74	

^a, b, c, d^—homogenous group.

## Data Availability

Not applicable.
